# Clinical audit of health promotion of vitamin D in one general practice

**DOI:** 10.1186/1447-056X-11-3

**Published:** 2012-04-02

**Authors:** Marjan Kljakovic, Cathy Davey, Rashmi Sharma, Divya Sharma

**Affiliations:** 1 Academic Unit of General Practice in the School of General Practice, Rural and Indigenous Health, at the Australian National University Medical School, PO Box 11 Woden, ACT 2606 Canberra, Australia; 2 Practice nurse at Isabella Plains Medical Centre, 9 Arakoon Crescent Isabella Plains, ACT 2905 Canberra, Australia; 3 General practitioner at Isabella Plains Medical Centre, 9 Arakoon Crescent, Isabella Plains, ACT 2905 Canberra, Australia; 4 General practitioner at Isabella Plains Medical Centre, 9 Arakoon Crescent, Isabella Plains, ACT 2905 Canberra, Australia

**Keywords:** Vitamin D, Health promotion, General practice, Clinical audit

## Abstract

**Background:**

The clinical audit of vitamin D health promotion in one Australian general practice was undertaken by measuring health service use and serum 25-hydroxyvitamin D levels in 995 patients aged 45 to 49 years.

**Findings:**

Over 3 years, 486 (51%) patients had a Medicare funded Health Assessment. More women (54%) were assessed than men (46%) p = 0.010. Mean 25-OHD was higher for men (70.0 nmol/l) than women (60.3 nmol/l) p < 0.001. More patients had their weight measured (50%) than 25-OHD tested (28%).

Among 266 patients who had a 25-OHD test, 68 (26%) had normal levels 80+ nmol/l, 109 (41%) were borderline 51-79 nmol/l, and 89 (33%) were low < 51 nmol/l. Mean 25-OHD was higher in summer (73.7 nmol/l) than winter (54.7 nmol/l) p < 0.001. Sending uninvited written information about 25-OHD had no effect on patients' subsequent attendance.

**Conclusions:**

Health promotion information about vitamin D was provided to 50% of a targeted group of patients over a one-year period. Provision of this information had no effect on the uptake rates of an invitation to attend for a general health assessment.

## Findings

The RACGP (Royal Australian College of General Practitioner) Red Book provides specific recommendations on vitamin D and sunlight exposure in health promotion[1]. This clinical audit was initiated when one GP (general practitioner) at the IPMC (Isabella Plains Medical Centre) observed that many patients appeared to be deficient in serum vitamin D. The aim of this clinical audit was to evaluate how IPMC managed its health promotion surrounding vitamin D.

## Method

### Practice setting

IPMC has 10 full-time equivalent GPs, four practice nurses, and five allied health workers who care for a practice population of 19,417 patients. The audit occurred in two parts over a three-year period between November 2006 and December 2009. The selection criteria included all patients registered with IPMC electronic health records and eligible for a Health Assessment funded through Medicare Item Number 717 (A policy introduced by the commonwealth government on 1st November 2006 meant for patients aged 45 to 49 years to consult for reasons of health promotion in general practice).

### Part 1 Audit

described the characteristics of patients aged 45-49 years who consulted the practice under Item Number 717 between November 2006 and October 2008.

### Part 2 Audit

measured the impact of written advice on patients who consulted IPMC for health promotion between October 2008 and December 2009. Patients were sent a letter inviting them to consult with IPMC. Half were randomly selected to receive the invitation only, the other half received the invitation letter as well as specific information contained in a Cancer cosmetics pamphlet [2] on the role of vitamin D and how to obtain a blood test.

The variables measured in this audit were the number of people who attended IPMC under Item Number 717 and their serum 25-OHD (25-hydroxyvitamin D) levels, the serum biomarker of vitamin D. Patients who are subsequently found to have a low 25-OHD level are routinely retested at six months intervals. The local laboratory determined the normal range for 25-OHD was 51 nmol/l to 140 nmol/l: A low level was defined as < 51 nmol/l 25-OHD, 51-79 nmol/l was defined borderline, and 80+ nmol/l was defined normal. The season in which the test was taken was also measured). Other variables included gender, weight, BMI, waist circumference, and the level of activity patients reported they performed in daily life. (Ranked on a 14-point Likert scale where 0-points was no daily physical activity to 14-points was 60 minutes of activity daily at a very strenuous level).

Data were coded in excel and analysed in PASW Statistics 18™ comparing differences between categories using the non-parametric chi squared test where appropriate.

Ethical approval was not sought for this audit because it met the National Health and Medical Research Council's criteria for exemption [3], namely the audit was undertaken with the consent of IPMC and patients were unlikely to suffer any burden or harm. Only anonymous data were collected for this audit and stored at the general practice.

## Results

### Part 1 of Audit

Of 955 patients audited, 486 (51%) had a Medicare funded Health Assessment and significantly more women (54%) had this assessment than men (46%) (316 women versus 170 men, Pearson Chi-Square = 6.57, df = 1, p = 0.010). Among the 469 (49%) patients who did not have a Medicare funded Health Assessment, significantly more men (54%) did not have this assessment than women (46%) (202 men versus 267 women, Pearson Chi-Square = 6.57, df = 1, p = 0.010).

There were 474 patients (50%) who had their weight measured; 473 (50%) had their BMI score calculated; 427 (45%) had their waist measured; 432 (45%) had their activity score measured; And 266 (28%) had a 25-OHD test. Table 1 shows males were significantly heavier and had larger waists than women, but no difference in BMI or activity scores than women. Women had significantly lower 25-OHD than men, even though more women had a 25-OHD test taken than men (32% versus 21%, Pearson Chi-Square = 13.28, df = 1, p < 0.001).

**Table 1:** Gender comparison of weight, waist, BMI, Activity score, and serum 25-hydroxyvitamin D (25-OHD) levels in 955 patients aged 45 to 49 years in one general practice in Canberra

Variable	Male				Female				*t *test	P value
			
	Minimum	Maximum	Mean	SD	Minimum	Maximum	Mean	SD		
Weight (Kilograms)	55	125	90.0	14.2	47	143	78.34	17.8	8.5	0.000
Waist (centimetres)	72	136	96.9	12.6	26	127	86.88	14.2	8.3	0.000
BMI* score	20	43	27.4	4.4	21	53	28.08	6.7	0.1	0.471
Activity score	0	8	3.9	2.0	0	10	3.09	2.2	-	0.500*
25-OHD level (nmol/l)	10	141	74.0	28.8	12	228	60.3	26.3	3.8	0.000

BMI = Body mas index (kg/m^2^) Reference range BMI of ≥ 25 conveys increased risk [1]

Waist reference range ≥ 94 cm in males and ≥ 80 cm in females conveys increased risk [1]

* Mann-Whitney *U *test

Among 486 patients who had a Health Assessment, 220 patients (45%) did not have a 25-OHD test, 207 patients (43%) had one test, 46 patients (9.5%) had two 25-OHD tests, and 13 patients (3%) had three 25-OHD tests.

Among the 266 patients who had 25-OHD tests, 89 patients (33%) had low 25-OHD of < 51 nmol/l, 109 patients (41%) had borderline 25-OHD of 51-79 nmol/l, and 68 patients (26%) had normal 25-OHD of 80+ nmol/l. Table 2 shows that 59 patients (66%) had a follow up 25-OHD test six months later. Among the 56 patients who had low 25-OHD at initial assessment, 19 patients (34%) remained at a low level at follow up, 32 patients (57%) changed to borderline, and 5 patients (9%) changed to normal.

**Table 2:** The number of patients who had initial and six-month follow-up serum 25-hydroxyvitamin D (25-OHD) taken as part of their Health Assessment in general practice *n *= 266 patients

Number of patients with initial 25-OHD levels*		%	Follow up 25-OHD levels at six months				
					
			Low	Borderline	Normal	Total followed	%
Low	89	33.5%	19	32	5	56	63%
Borderline	109	41%	1	1	1	3	3%
Normal	68	26%	-	-	-	0	0%

*Low 25-hydroxyvitamin D = < 51 nmol/l, Borderline 25-hydroxyvitamin D = 51-79 nmol/l, Normal 25-hydroxyvitamin D = 80+ nmol/l

Table 3 shows a seasonal variation of when the 25-OHD tests were taken in 266 patients with 34% taken in autumn, 14% in winter, 20% in spring, and 31% in summer. The variation among the 89 patients who had low 25-OHD differed with 31% taken in autumn, 22% in winter, 33% in spring, and 13% in summer.

**Table 3:** Seasonal variation of serum 25-hydroxyvitaminin D (25-OHD) in 266 patients from one general practice in Canberra

			Number		
			
Season	*n*	Mean 25-OHD level (nmol/l)	< 51 nmol/l	51-79 nmol/l	80+ nmol/l
Autumn	91	66.1	28	42	21
Winter	38	54.7	20	11	7
Spring	54	53.8	29	18	7
Summer	83	73.7	12	38	33

### Part 2 of Audit

Of the 584 patients who were sent general health advice in the mail, 50% were randomly selected to receive additional written information about vitamin D, and 50% were not. Part 2 Audit resulted in an additional 115 patients having a Health Assessment (20% of total). There was no difference in the proportion of patients who had a Health Assessment and received specific written information about vitamin D compared to those who did not (21% versus 18%, Pearson Chi-Square = 0.592, df = 1, p = 0.442).

## Discussion

This audit revealed that after three years of work IPMC had attempted a Health Assessment of the vitamin D status in 51% of patients aged 45 to 49 years. Although half of these patients had their weight measured, only 28% had a test to measure 25-OHD.

The audit revealed a bias towards testing patients who had low or borderline 25-OHD. This suggests that the 25-OHD test was not used as a screening tool; rather it was used for case finding. Knowledge of a patient's low 25-OHD appeared to have little impact on changing the patient's level. Only 8% of such patients were subsequently shown to revert back to normal levels.

The overall mean 25-OHD level (74.0 nmol/l) found in this audit was similar to the 76.9 nmol/l mean level found in a group of Adelaide residents[4] and higher than the 56.8 nmol/l mean level found in a recent study of adult Aboriginal Australians[5]. Furthermore, the audit confirmed the known seasonal variation in 25-OHD [5] by finding a high proportion of 25-OHD tests taken in summer compared to other seasons and a comparatively low proportion of patients with low levels of 25-OHD in summer. In our audit, we did not measure the amount of time patients spent outside. Therefore we can only speculate that the paradoxical relationship between a high proportion of testing undertaken in summer and a low proportion of patients with low levels of 25-OHD in summer is a consequence of patient behaviour - perhaps patients spend less time outside in response to high temperatures in the ACT in the summer months.

The audit revealed a gender imbalance: Women were more likely to have a health assessment than men and consequently were more likely to have a test. However, women were also found to have significantly lower 25-OHD level than men. Furthermore, men were significantly heavier and had larger waists than women, but no difference in BMI or activity scores. These differences suggest that men and women need different practice policies for health promotion.

The audit demonstrated that sending uninvited health promotion information to patients had no effect on subsequent attendance for health promotion in this practice. New health promotion strategies are needed. For example, sending a newsletter to the whole practice population, or working in conjunction with the local media, might stimulate more people to consider the relationship between vitamin D and the amount of sunlight exposure they experience. The RACGP guidelines for preventive activities in general practice list the strategies that Australian general practice might undertake for health promotion and mentions vitamin D [1]. The guidelines do not mention how practices might vary their strategies in response to specific characteristics such as the gender profile of the general practice.

The limitations of this clinical audit include the bias of using only one general practice, the non-random selection of patients, the local laboratory determined the normal serum 25-OHD reference range, and the measurement error inherent in undertaking an audit. (In Australian general practice patients may go elsewhere to manage their low 25-OHD and there are limited mechanisms to ensure patients comply on follow up of low-test results). Finally we did not measure the amount of sunlight exposure in patients.

In summary, a considerable amount of work was undertaken by IPMC over three years resulting in half the target group of patients receiving health promotion, just over a quarter had the appropriate blood test, and none were influenced by uninvited written health information on vitamin D. The audit taught IPMC that men and women need different policies for health promotion on the association between 25-OHD levels and time spent outside in summer.

## Abbreviations

GP: general practitioner; IPMC: Isabella Plains Medical Centre; 25-OHD: 25-hydroxyvitamin D; RACGP: Royal Australian College of General Practitioners.

## Competing interests

The authors declare that they have no competing interests.

## Authorship

MK conceived of the study, participated in its design and statistical analysis, and helped to draft the manuscript. CD coordinated with IPMC and collected all data. RS and DS supervised CD and helped with coordinating with IPMC. All authors read and approved the final manuscript.
